# Surveillance of Chronic Non-communicable Diseases: thoughts on the role of national health surveys of Brazil

**DOI:** 10.1590/SS2237-9622202200013.especial

**Published:** 2022-07-18

**Authors:** Sheila Rizzato Stopa, Célia Landmann Szwarcwald, Max Moura de Oliveira, Silvânia Suely Caribé de Araújo Andrade

**Affiliations:** 1 Independant researcher, Epidemiology, Brasília, DF, Brazil; 2 Fundação Oswaldo Cruz, Instituto de Comunicação e Informação Científica e Tecnológica em Saúde, Rio de Janeiro, RJ, Brazil; 3 Universidade Federal de Goiás, Departamento de Saúde Coletiva, Goiânia, GO, Brazil; 4 Ministério da Saúde, Departamento de Ações Programáticas e Estratégicas, Brasília, DF, Brazil

From the mid-twentieth century, several changes took place in global demographic, nutritional and epidemiological patterns.^1^ The processes of social and economic development, improvements in environmental conditions and public health, and advances in medicine and health care contributed to the sharp drop in the fertility rate, to a marked reduction in infant and childhood mortality, and to the increase in life expectancy.^2^ With the ageing of the global population and changes in the morbidity and mortality profile, noncommunicable diseases (NCDs) have become the most relevant health problem in most countries.^3^

In view of the acknowledgment that the risk of developing a NCD can be significantly reduced through the adoption of public policies that support better living and health conditions, some strategies were established in order to increase people's quality of life and, thereby, improve the health of populations.^4^ As part of the Sustainable Development Goals (SDGs), most public health actions have focused on health promotion, encouraging healthy behaviors and the reduction of risk factors.^5^ Delaying the onset of complications and disabilities through early detection and providing quality care to alleviate the severity of chronic problems have also been considered fundamental strategies.^6^

In Brazil, NCDs have accounted for a high number of deaths before the age of 70 and loss of quality of life with ageing, generating disabilities and a high degree of limitation in work and leisure activities. National studies based on data from 1990 to 2017 indicate that NCDs are responsible for more than 70% of deaths and a large proportion of unhealthy life years.^7^ ^,^ ^8^ The expansion of primary care throughout the national territory and the National Policy for Health Promotion (PNPS), introduced in the mid-2000s, were milestones in the implementation of actions related to the control of arterial hypertension and diabetes, and to the prevention of NCDs, by means of strategies aimed at promoting physical activity, healthy eating habits, weight control, and at preventing smoking and alcohol abuse.^9^

In this context, it is relevant and opportune to discuss the implementation of NCDs surveillance in Brazil and at subnational levels.

## Surveillance of non-communicable diseases in Brazil

Aiming to monitor the magnitude and spatio-temporal distribution of NCDs, associated risk factors and the health care provided to the chronically ill, in order to support management, NCDs surveillance is carried out by means of secondary data from health information systems and primary data collected in health surveys.^10^

Among such information systems, the following stand out: the Hospital Information System of the Brazilian National Health System (SIH-SUS), which contains data on diagnoses and hospital admission expenses,^11^ and the Mortality Information System (SIM), which collects information on deaths in all Brazilian municipalities and allows monitoring premature mortality due to NCDs.^12^ A survey carried out by the Health Surveillance Secretariat of the Ministry of Health (SVS/MS), in 2018,^13^ aimed to identify the existing structure, as well as the development of NCDs epidemiological surveillance actions in state and municipal health secretariats in the state capitals (SES and SMS Capitals), found that the main sources of information for NCDs surveillance were SIM and SIH, in line with a previous study.^14^ Both systems are essential for the surveillance of NCDs, as they provide an overview of the epidemiological situation of deaths and hospitalizations in the Brazilian population. Although there are differences in coverage and quality of information, the analysis of data arising from these systems can support interventions at different levels of geographic disaggregation (federal, state and municipal).^15^

However, in order to strengthen the surveillance of NCDs, besides the need to invest in improving the coverage and quality of secondary data related to hospital mortality and morbidity, it is necessary to encourage the conduction of frequent and regular health surveys that enable monitoring the prevalence of NCDs, the adoption of healthy behaviors and the reduction of habits that are harmful to health, as well as the adequacy of health care from the perspective of the user.^16^

## Brazilian national health surveys

Among the surveys conducted by the Ministry of Health in terms of the surveillance of NCDs, which support the monitoring of the 2011-2022 Strategic Action Plan for Addressing Chronic Noncommunicable Diseases (NCDs) in Brazil, and the responses to the Regional^17^ and Global^18^ Plans, as well as the SDGs,^19^ the following stand out: the Chronic Disease Risk and Protective Factors Surveillance Telephone Survey (VIGITEL), the National Adolescent School-based Health Survey (PeNSE) and the National Health Survey (PNS).

VIGITEL, the most sustainable health survey in the country, presents the evolution of important life habits of Brazilian adults living in the capitals and was essential in monitoring the goals set by managers from different areas of the Ministry of Health and other entities of the federation over the past two decades.^20^ PeNSe, carried out in partnership with the Brazilian Institute of Geography and Statistics (IBGE), investigates the risk and protection factors for NCDs in schoolchildren aged 13 to 17 years and makes it possible for the education and health sectors to articulate actions directed to this group.^21^

The PNS, the subject of this special edition of the journal *Epidemiologia e Serviços de Saúde* (RESS), is the largest health survey ever carried out in Brazil and provides a portrait of the living and health conditions of the country residents.^22^ Its design, specifically elaborated to provide estimates of health indicators and to enable analyses at various geographic levels and according to socioeconomic and demographic characteristics of individuals, makes it possible to establish health priorities that are relevant for management.^23^ Information from the two editions of the PNS, carried out in 2013 and 2019, provide an overview of the main NCDs and their associated risk factors in Brazil and bring important elements to support the surveillance of NCDs.

The PNS data are available to the public, and prior authorization to use the information is not required. The health indicators, selected by the Ministry of Health as being the most important for management, are available on the IBGE website (https://bit.ly/38DaRNd), for Brazil, large regions, Units of the Federation, state capitals, rural/urban area of residence and according to the sociodemographic characteristics of Brazilians.^24^ Additionally, in order to expand access to information and health indicators, Fundação Oswaldo Cruz (Fiocruz), through the Institute of Scientific and Technological Communication and Information in Health and in partnership with SVS/MS, created a panel with data from the two editions of the PNS, available at https://www.pns.icict.fiocruz.br.^25^ It is possible to access and download the estimates of several indicators, view them in tables, graphs or maps, for all the socioeconomic and geographic disaggregation established for both of the PNS.

It is understood that it would be of great importance for this strategy to be extended to other surveys conducted by the Ministry of Health, in order to facilitate and expand the use of information available to health managers and staff so that they can be used in a timely manner in the (re)direction of actions to tackle NCDs. 

A challenge that still needs to be faced in Brazil is obtaining reliable information about NCDs and their risk factors within the scope of the SUS Information Systems, and which can be accessible to health managers and workers. In these terms, the e-SUS strategy^25^ is a possibility, as it encompasses data from the Primary Care Registry, whose function, in turn, is to aggregate socioeconomic and health characteristics of the population of a given territory, including aspects of interest for the surveillance of NCDs,^26^ mainly at levels of disaggregation that will not be represented in national surveys. However, it is known that there are difficulties, especially for SES and SMS NCDs surveillance workers, in using information solely from this system, since part of its access is controlled and restricted.^26^ In fact, a survey carried out by the SVS/MS pointed to low use of the e-SUS to carry out surveillance of NCDs.^13^

However, it is not just information gaps that interfere with NCDs surveillance at subnational levels. In 2018, more than 20% of SES or SMS Capitals did not have the NCDs surveillance area incorporated into their institutional organizational charts; the average number of professionals involved in the composition of surveillance was six for SES and three for SMS Capitals.^13^ The situation of smaller municipalities must probably be even more precarious.

Conducting large health surveys, such as those briefly presented here, is a difficult task. It takes time to plan, besides the engagement of various areas of the Ministry of Health for its execution and funding, collaboration of researchers and articulation between bodies and academic institutions. Given that NCDs continue to be the main causes of premature mortality in the country, ensuring the sustainability and periodicity of research that encourage discussion and review of policies to control NCDs should be a priority for SUS. Additionally, to activate and improve the surveillance of NCDs at different geographic levels, it is necessary for the Ministry of Health to guide its SES and SMS Capital counterparts on the use of available information, so that the instrumentalization of management is based on scientific evidence.
