# Erratum

**DOI:** 10.1590/SS2237-96222022000020.especial

**Published:** 2022-11-25

In the article “Surveillance of Chronic Non-communicable Diseases: thoughts on the role of national health surveys of Brazil”, doi: 10.1590/SS2237-9622202200010.especial, published on Epidemiology and Health Services, 31(nspe1):e20211048, 2022, in the page 1: 

Original text:

10.1590/SS2237-9622202200010.especial

Corrected text:

10.1590/SS2237-9622202200013.especial

